# A Rhizosphere-Associated Symbiont, Photobacterium spp. Strain MELD1, and Its Targeted Synergistic Activity for Phytoprotection against Mercury

**DOI:** 10.1371/journal.pone.0121178

**Published:** 2015-03-27

**Authors:** Dony Chacko Mathew, Ying-Ning Ho, Ronnie Gicaraya Gicana, Gincy Marina Mathew, Mei-Chieh Chien, Chieh-Chen Huang

**Affiliations:** 1 Department of Life Sciences, National Chung Hsing University, Taichung, Taiwan, R. O. C; 2 Department of Plant Pathology, National Chung Hsing University, Taichung, Taiwan, R. O. C; 3 School of Biosciences, Mar Athanasios College for Advanced Studies (MACFAST) BIOCAMPUS, Tiruvalla, Kerala, India; CEA-Saclay, FRANCE

## Abstract

Though heavy metal such as mercury is toxic to plants and microorganisms, the synergistic activity between them may offer benefit for surviving. In this study, a mercury-reducing bacterium, *Photobacterium* spp. strain MELD1, with an MIC of 33 mg . kg^-1^ mercury was isolated from a severely mercury and dioxin contaminated rhizosphere soil of reed (*Phragmites australis*). While the whole genome sequencing of MELD1 confirmed the presence of a *mer* operon, the mercury reductase *MerA* gene showed 99% sequence identity to *Vibrio shilloni* AK1 and implicates its route resulted from the event of horizontal gene transfer. The efficiency of MELD1 to vaporize mercury (25 mg . kg^-1^, 24 h) and its tolerance to toxic metals and xenobiotics such as lead, cadmium, pentachlorophenol, pentachloroethylene, 3-chlorobenzoic acid, 2,3,7,8-tetrachlorodibenzo-p-dioxin and 1,2,3,7,8,9-hexachlorodibenzo-p-dioxin is promising. Combination of a long yard bean (*Vigna unguiculata* ssp. *Sesquipedalis)* and strain MELD1 proved beneficial in the phytoprotection of mercury *in vivo*. The effect of mercury (Hg) on growth, distribution and tolerance was examined in root, shoot, leaves and pod of yard long bean with and without the inoculation of strain MELD1. The model plant inoculated with MELD1 had significant increases in biomass, root length, seed number, and increased mercury uptake limited to roots. Biolog plate assay were used to assess the sole-carbon source utilization pattern of the isolate and Indole-3-acetic acid (IAA) productivity was analyzed to examine if the strain could contribute to plant growth. The results of this study suggest that, as a rhizosphere-associated symbiont, the synergistic activity between the plant and MELD1 can improve the efficiency for phytoprotection, phytostabilization and phytoremediation of mercury.

## Introduction

The onset of industrial revolution have resulted in severe and widespread contamination of land and underground water by toxic heavy metals such as mercury that cannot be degraded but can only be reduced to a less toxic form. This contamination is primarily as a result of fossil fuel combustion, chloralkali industry and usage of Hg for gold extraction. Mercury is a toxic metal that accumulates through the food web and Hg pollution causes global health problems [1]. Chronic exposure to mercury can cause detrimental effects to human health like neurosensory disorders, immune malfunction, irregular blood pressure, myocardial infarction and renal toxicity [2].

In the environment, mercury may be present in concentrated “hot spots” or dispersed over a large area. Often, contamination is initially suspected on the basis of historical land use (*e*.*g*. in the production of primary batteries). Since, environmental cleanup rates of mercury contaminated sites are low, various *in-situ* mercury treatment technologies like soil vapour extraction [3], reactive walls, *In-situ* leaching [4], chemical immobilization [5], water interceptors, wetlands and phytoremediation [6] are currently used. The high cost and technical difficulty of mercury removal from the environment has provided impetus for studies in which plants are used as an inexpensive means to clean up the environmental. Although plants and their associated microorganisms can degrade a wide range of compounds within the plant rhizosphere [7], seemingly the simplest solution to this problem would be to mix beneficial soil bacteria with appropriate biodegradative or reducing capabilities, possibly as a soil drench to the roots of seedlings planted at the contaminated sites [8]. Bacteria that are associated with plant roots exert beneficial effects on plant development and are referred to as plant growth- promoting rhizobacteria (PGPR). They are noted to colonize plant roots and simultaneously act as biofertilizers and as antagonists (biopesticides) of prominent root pathogens, including other bacteria and fungi [9].

Phytoremediation, the use of plants to remove, partition, or sequester toxic metals and other hazardous substance from the environment [10] is a relatively new approach for the cleanup of polluted environments. The plants that are relatively tolerant to various environmental contaminants often remain underdeveloped, or grow slowly in the presence of toxic metals. The rhizosphere is a rich niche of microbes and should be explored for potential PGPR, which can be useful in developing bio-inoculants for enhancement of growth and yield of crop plants. In addition to their agricultural usefulness, there are potential benefits in environmental applications like their ability to fix atmospheric nitrogen, solubilize phosphorus and iron, and enhance production of plant hormones. Additionally, they improve the plant tolerance to drought, high salinity, heavy metal and pesticide toxicity. A technology combining plant and microorganisms, mainly plant growth promoting bacteria (PGPB) inoculated into the plant roots, may have a synergistic action, leading to improved plant growth [11]. In recent studies it was shown that some bacterial strains could assist in the phytoprotection and phytodegradation of various toxic compounds [12]. Phytoremediation of contaminated lands assisted by PGPR is a promising field yet to be commercially applied despite the large volume of knowledge on that topic. On the other hand, bacterial metal resistance has been described as a necessity for plant associated bacteria in contaminated environments [13]. Some bacteria have a mercury resistance mechanism, based on a group of genes located in the *mer* operon. The *mer* genes, in these bacteria are often located in plasmids or transposons but can also be found in chromosomes [14]. The major bacterial mechanism of mercury resistance includes the uptake and transport of Hg^2+^ by the periplasmic protein MerP, MerF a mercuric ion transport protein [15] and the inner membrane protein MerT. The mercury reductase MerA allows bacteria to detoxify Hg^2+^ into volatile metallic mercury by enzymatic reduction to much less toxic volatile Hg° [16]. Certain Gram-negative and positive bacteria have the ability to reduce Hg^2+^ to Hg° using the enzyme mercury reductase [17]. MerR is the activator or repressor of the transcription of *mer* genes in presence or absence of mercury ions [18]. During mercury stress condition the transcriptional activator MerR triggers the expression of the structural *mer* genes [19].

Studies related to the potential use of plants from Leguminosae family used in phytoremediation or phytostabilization of heavy metals is rare and few evidences suggest that plants such as *Lupinus albus* [20], *Vicia faba* [21] and *Trifolium repens* [22] were used. Based on this, the current study was initiated to explore the potential of yard-long bean in phytoremediation of mercury. Legumes are effective in soil restoration and in preparation for colonization of other species and because of this innate fertilizing function; they are regarded as pioneer species in seral succession [23– 24]. The soil restoration with legumes under limiting environmental conditions requires a selection of rhizosphere bacteria that are able to resist the high toxicity of heavy metals in this case, the high mercury concentration in the soil.

To screen the potential candidates for rhizosphere-associated symbiont for phytoremediation, soils firmly adhered to dominant plants growing in the heavy metal contaminated An-shun site (Tainan, Taiwan), such as *Phragmites australis* and *Vetiveria zizanioides*, were used as the source for bacterial isolations. The An-shun factory produced chloroalkali and pentacholorophenol (PCP) over four decades, and the highest mercury concentration was 3,370 mg/kg while the worst dioxin concentration was 64,100,000 ng-I- Toxic Equivalency (TEQ) /kg in soil [25]. The objective of the present work aims to examine the impact of high concentrations of mercury on the growth of yard long bean inoculated with and without a rhizosphere-associated symbiont strain. In this paper we have shown the isolated *Photobacterium* strain possesses abilities to reduce high concentrations of mercury ion as well as having plant growth promoting properties, hence we assess the potential of yard long bean to be a suitable candidate for phytostabilization and phytoremediation of mercury contaminated soil.

## Materials and Methods

### Ethical statement

The entire sample sites were distributed in the An-Shun site belonging to the China Petrochemical Development Corporation (CPCD-ASS) and permission were approved from CPCD and the Tainan government. The field studies did not involve endangered or protected species. The coordinates of An-shun sample sites were at Lat. 23° 3′ 30” N, Long. 120° 8′ 9” E.

### An-shun factory site description

The An-shun factory site (CPDC-ASS) is located in the Tainan city, on the south-west coast of Taiwan [26, 27]. The mean temperatures in summer and winter are 29°C and 21°C, respectively, whereas the annual precipitation averages 16.7 cm. From 1942 to 1982, the An-shun factory produced chloro-alkali and pentacholorophenol (PCP). The analysis of the soil showed a high concentration of mercury, dioxins, lead and cadmium [28– 29]. Despite the toxic environment, grasses like *Phragmites australis* and *Vetiveria zizanioides* were found to be dominant plants growing in this area. The mean daily temperature of the operational time was 22 ± 10°C during the sample collection.

### Isolation of plant root colonizing bacteria

Healthy *Phragmites australis* plants were collected from heavy-metal contaminated An-shun factory site in Tainan, Taiwan. For the isolation of rhizosphere bacteria, the clumps of loosely soil adhering to the roots were removed and roots with soil firmly adhered to them were suspended in sterile 0.85% NaCl. About 10 g of rhizosphere soil was transferred to a 250 ml Erlenmeyer flask containing 100 ml of sterile distilled water, and the flask was shaken at 150 rpm for 30 min. Serial dilutions were immediately prepared, and were spread plated on Luria Bertani (LB) and M9 minimal media plates incubated at 28°C for 24 hours [30]. Luria Bertani medium was used according to the isolation of soil bacteria followed by Ueno et al [31].

For the isolation of endophytic bacteria the roots were taken out from these suspensions, surface sterilized with 70% ethanol for 5 min, followed by 1% commercial bleach Clorox (Oakland, California) and a 0.01% Tween 20 solution for 1 min, and then washed thrice in double distilled water [32]. Root materials (0.2 g) were homogenized using mortar and pestle, serially diluted and plated on LB plate and incubated at 36 h for 28°C.

### Screening for heavy metal resistance

The selected bacterial isolates were tested for their resistance to heavy metals by agar dilution method [33]. Freshly prepared LB plates amended with soluble salts of heavy metals like mercury, lead and cadmium of varying concentrations determined by the average concentration of the heavy metals in the An-shun site soil were inoculated with bacterial strain isolated from *Phragmites australis*. The concentrations of the mercury (Riedel-de Haën, Switzerland) used were 20, 25, 30, 35, 40, 45 mg. kg^-1^, for lead and cadmium (Riedel-de Haën, Switzerland) 100, 150, 200, 250, 300 mg. kg^-1^. The isolated strains were also screened for resistance to 2, 4, 6, 8 and 10 mg. kg^-1^ for pentachlorophenol (PCP) (Sigma, USA), pentachloroethylene (PCE) (Sigma, USA) and 3-chlorobenzoic acid (CBA) (Sigma, USA), 1, 2, 3 mg. kg^-1^ of 2,3,7,8-tetrachlorodibenzo-p-dioxin (2,3,7,8-TCDD) (Sigma, USA) and 1, 2, 3 mg. kg^-1^ of 1,2,3,7,8,9- hexachlorodibenzo-p-dioxin (HXCDD) (Sigma, USA). The maximum tolerable concentrations (MTC) [34] of heavy metal was designated as the highest concentration of heavy metal that allows growth after 2 days i.e., 48 hrs [35]. The heavy metal resistant screened cultures were characterized based on their taxonomy and 16S rRNA sequencing.

### Molecular characterization by 16S rRNA genes

A 16S rRNA gene phylogenetic tree were constructed for the heavy metal screened strains using the neighbor—joining distance method with MEGA 6.06 software [36]. The Bootstrap percentages were calculated using 1000 repetitions. Total microbial DNA was isolated from the bacterial cultures using the Easy Tissue and Cell Genomic DNA Purification Kit, (Genemark, Taichung, Taiwan) according to the manufacturer’s instructions. The bacterial 16S rRNA sequences were amplified using the universal primer E8F (5^’^AGAGTTTGATCCTGGCTCAG) and U1510R (5^’^ GGTTACCTTGTTACGACTT), and the PCR amplicons were ligated into the ‘yT and A’ cloning vector (Takara BIO INC, Japan). Transformation and blue and white colony selection were performed according to the protocol by Sambrook et al., 2001. The sequences were compared with reference 16S rRNA gene sequences in the GenBank using CLUSTAL X 1.83 [37] software and the strains were identified.

### MerA gene amplification

The mercury resistant isolated strains were analyzed for the *merA* gene, which is able to reduce Hg^2+^ to Hg° using the enzyme mercury reductase. The mercury resistance gene of the bacterial strains was identified by PCR using *merA* gene specific primers for Gram negative bacteria [38]. After total genomic DNA isolation, the following primers were used for PCR amplification of the *merA* gene: A1s-n.F (5’-‘TCCGCAAGTNGCVACBGTN-3’), A5-n.R (5’-ACCATCGTCAGRTARGGRAAVA-3’). The primers were specifically designed to amplify the conserved region in the pyridine nucleotide disulfide oxidoreductase dimerization domain of Tn*501 merA* gene in Gram negative bacteria. Each reaction mixture contained 0.1μl *Taq* DNA polymerase (USB, Cleveland), 1X PCR buffer, 0.2 mM dNTP’s, each primer at a concentration of 10 pM, and 50 ng of DNA. The thermocycling conditions consisted of a denaturation step at 95°C for 2 min, 29 amplification cycles of 95°C for 1 min, 55°C for 2 min, and 72°C for 3 min, and a final polymerization step of 72°C for 5 min with a Veriti Thermal cycler of Applied Biosystems 9600 (Veriti Thermal Cycler, U.S.A). PCR products were visualized on 0.8% agarose gel, and the products were purified. The presence of *merA* gene was confirmed by the detection of PCR amplicons of 285 bp band which is the conserved region in the pyridine nucleotide disulfide oxidoreductase dimerization domain of *merA* gene.

### Mercury reductase assay

To test the mercury reductase activity, the selected strains were grown in LB medium containing 25 mg. kg^-1^ of Hg for 24 h at 150 rpm (28°C). The initial concentration of Hg 25 mg. kg^-1^ was determined by the average concentration of Hg found in the An-shun site soil samples. The samples were analyzed in triplicates and the mercury reduction rates were analyzed in 24 h using Inductively Coupled Plasma Optical Emission Spectrometry (ICP-OES, Optima 5300DV, Perkin-Elmer Co., USA).

### Whole genome sequencing

*Photobacterium* species are bacterial species associated with marine organism, while MELD1 was isolated from plants and to understand the molecular mechanism of the plant- microbe interaction the whole genome sequencing was performed. 10ug of total DNA was sonicated by Misonix 3000 sonicator to the size ranging from 400 to 500bp. DNA sizing is checked by bioanalyzer DNA 1000 chip (Agilent Technologies). 1ug sonicated DNA was end-repaired, A-tailed and adaptor-ligated following the Illumina‘s Trueseq DNA preparation protocol.

The sequences generated went through a filtering process to obtain the qualified reads. ConDeTri [39] was implemented to trim or remove the reads according to the quality score. Cleaned and filtered nuclear reads were assembled *de novo* using Abyss [40]. Genome annotations were created in MAKER 2.00 [41] using a GeneMark [42] model trained for MELD1 via self-training. The resulting predictions were searched against NCBI non-redundant (nr) database by using BLASTP.

### Indole-3-acetic acid (IAA) production

To analyze if the isolated strains could contribute to plant growth, the IAA production was measured according to Sachdev et al [43]. An aliquot of 1 ml bacterial suspension was grown in LB broth with L-tryptophan (0.5 mg. ml ^-1^) for three days and then was transferred into a tube and mixed vigorously with 2 ml of Salkowski’s reagent. A pink color developed after 20 min incubation at room temperature, and the absorbance was measured at 530 nm. The IAA concentration was determined using a calibration curve of pure IAA as a standard following the linear regression analysis. To test the IAA production under laboratory conditions, the model plant *Arabidopsis thaliana* were used. The model plants were equally spaced in a ½ MS medium petri plate, and the bacteria were inoculated 2 cm away from the plantlet’s root. The growth of bacterial cultures was maintained to 0.7 O.D at 600 nm and at 28°C.

### Soil sampling for Gnotobiotic assay

The subsoil (10–30 cm) from the An-shun factory site was collected for the gnotobiotic assay. Collected soil were sieved through a 1-mm sieve to remove large stones, plant residues and soil fauna, and then homogenized to give a composite sample. The soil had an average Hg concentration of 27 mg. kg^-1^, pH of 8.0 and cation exchange capacity (CEC) of 3.5 mmol. kg ^-1^. According to percentages of sand (54%), silt (29%) and clay (19%) of the matrix collected, it could be classified as a sandy loam soil (USDA classification).

### Gnotobiotic assay with Vigna unguiculata

The mercury contaminated soil from An-Shun factory site were used in this experiment. The seeds were collected from local farmers in Kerala, India. Seeds were surface sterilized using 90% ethanol for 3 min and 3.5% sodium hypochlorite for 30 min. The strain, were grown to mid-log phase between 0.7 to 1.3 O.D at 600nm. The surface sterilized seeds were sown in plastic pots (top diameter, 21cm; bottom diameter, 14.5 cm; height, 19.5 cm) and were filled with approximately 5 kg of mercury contaminated soil collected from An shun factory site having an average mercury concentration of 27 mg. kg^-1^. The seeds were grown to a period of 21 days before they were inoculated with the selected strain. The experiments were performed in five replicates for the inoculated and the un-inoculated plant, which served as the control. Soil drench inoculation methods were used in this experiment [44], the surrounding soils of the 21 day old plantlets were drenched with approximately 2 ml of strain. The day/night temperatures were maintained approximately between 23/26°C. The crops were uprooted after 3 months and the growth parameters such as root length, seed number, leaf number, dry weight and fresh weight were recorded and compared to the control plant. Before further analysis, the samples were surface sterilized using 1 mM HCl to remove the heavy metal particle that adhered to the exterior surface of the plant.

## Results

### Isolation and characterization of endophytes and rhizobacteria

**Fig 1:**
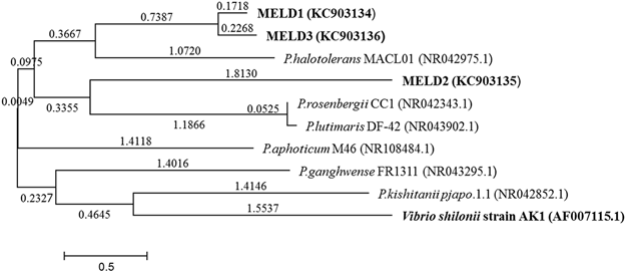
16s rRNA sequence analysis of three *Photobacterium* strains. The evolutionary history was inferred using the Neighbor-Joining method. The evolutionary distances were computed using the Maximum Composite Likelihood method.

A total of 64 isolates, of which 29 were from the surface sterilized roots and 35 from the rhizosphere of the reed *Phragmites australis*, a dominant plant species on An-shun factory contaminated site were screened. Out of the 64 isolates, 3 heavy metal resistant strains were sequenced by 16S rRNA analysis and the isolates were identified as *Photobacterium*, a Gram negative bacteria. The three *Photobacterium* strains isolated from the reed were named as MELD1, MELD2 and MELD3, of which MELD1 was isolated from the surface sterilized root and MELD2 and MELD3 from the rhizosphere. A phylogenetic tree was constructed using the 16S rRNA gene sequences from the bacterial isolates and similar sequences (Fig. 1). The phylogenetic tree shows that there is a high sequence homology between the MELD1 and MELD3 as compared to MELD2 and the degree of homology between MELD1 and MELD3 was approximately around 99% and with MELD2 was 98% sequence homology. MELD1 and MELD3 however were evolutionary closer to *P*.*halotolerance* (NR042975) and MELD2 was evolutionary closer to *P*.*rosenbergii* CC1.

Since the strains were isolated from an environment contaminated with heavy metals, they were assayed for metal tolerance. The mercury tolerance assay showed that out of the three strains MELD1 was tolerant the most tolerant to 35 mg. kg^-1^ Hg. Furthermore, MELD1 was observed to have higher tolerance to Cd, Pb, PCP, PCE, 3-CBA, TCDD and HXCDD as compared to MELD2 and MELD3 (Table 1 and Table 2).

**Table 1:** Maximum tolerable concentration for Lead, Cadmium and Mercury.

Strains	Lead (μg/ ml)					Cadmium (μg/ ml)			Mercury (μg/ ml)				
	100	150	200	250	300	100	150	200	20	25	30	35	40
MELD1	^+^	^+^	^+^	^+^	^-^	^+^	^+^	^-^	^+^	^+^	^+^	^+^	^-^
MELD2	^+^	^+^	^+^	^-^	^-^	^+^	^-^	^-^	^+^	^+^	^+^	^-^	^-^
MELD3	^+^	^+^	^+^	^+^	^-^	^+^	^-^	^-^	^+^	^+^	^-^	^-^	^-^

^+^ represent growth

^-^ represents no growth.

**Table 2:** Maximum tolerable concentration for Pentachloroethylene, 3-Chlorobenzoic acid and Pentachlorophenol.

Strains	PCE (μg/ ml)				3-CBA (μg/ ml)			PCP (μg/ ml)					TCDD (μg/ ml)				HXCDD (μg/ ml)			
	2	4	6	8	2	4	6	2	4	6	8	10	1	2	3	4	1	2	3	4
MELD1	+	+	+	+	+	+	-	+	+	+	+	-	+	+	+	-	+	+	+	-
MELD2	+	+	+	-	+	-	-	+	+	+	-	-	ND	ND	ND	ND	ND	ND	ND	ND
MELD3	+	+	+	+	+	+	+	+	+	-	-	-	ND	ND	ND	ND	ND	ND	ND	ND

PCE = Pentachloroethylene

3-CBA = 3-Chlorobenzoic acid

PCP = Pentachlorophenol

TCDD = 2,3,7,8- tetrachlorodibenzo-p-dioxin.

HXCDD = 1,2,3,7,8,9-hexachlorodibenzo-p-dioxin

^+^ represent growth

^-^ represents no growth

ND = No data.

### Identification and classification of merA gene and its mercury reductase activity

**Fig 2:**
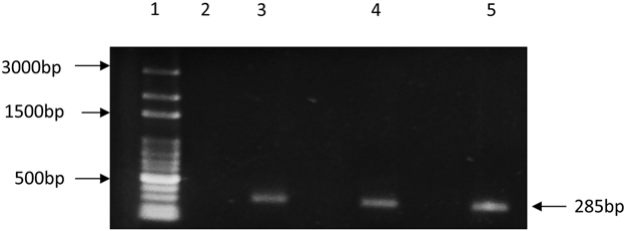
PCR detection of 285 bp conserved region of merA genes of MELD1, MELD2 and MELD3. Lane 1: 100bp DNA Ladder, Lane 2: No DNA control, Lane 3: MELD1, Lane 4: MELD2, Lane 5: MELD3.

The PCR amplification of the *merA* gene was the most compelling evidence to indicate the presence of *merA* gene. The presence of *merA* gene was confirmed by the detection of 285 bp band of conserved region in the pyridine nucleotide disulfide oxidoreductase dimerization domain (Fig. 2). Using Blast X, the conserved sequence showed 100% similarity to the *merA* sequence of *Vibrio shilonii* (ZP_01865594).

**Fig 3:**
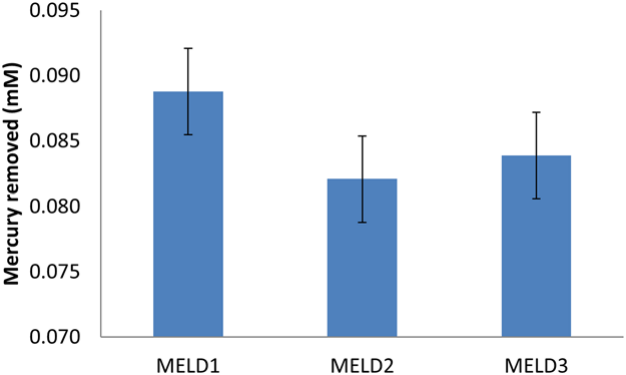
MELDI, MELD2 and MELD3 were grown in LB medium having a known concentration of 25 mg. kg^-1^ HgCl_2_. Mercury/MA- 2000 (AAS) was used to detect the mercury reductase activity of the strains. Bars plot mean ± SD of three replicate experiments.

The three *Photobacterium strains* were able to volatilize elevated levels of mercuric chloride from a high concentration of HgCl_2_. The mercury reductase activity of MELD1, MELD2 and MELD3 were noted for a period of 24 h with concentration of 25 mg. kg^-1^respectively. This concentration was used as a standard concentration, as it was determined all the 3 strains could grow at this concentration. The strain MELD1 was able to reduce 96% of Hg to a residual concentration of 0.90 mg. kg^-1^. This result suggested that although all the three strains could reduce the Hg^2+^ to Hg^0^, MELD1 significantly enhanced the reduction of Hg^2+^ in a controlled environment (Fig. 3).

### Whole genome sequencing of MELD1

**Fig 4:**
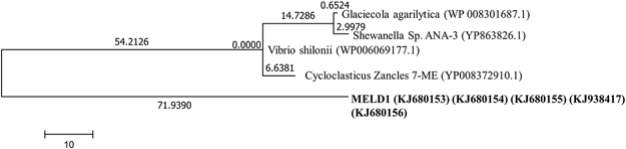
*mer* operon phylogenetic tree was constructed using the Maximum Likelihood method based on the JTT matrix-based model.

**Fig 5:**
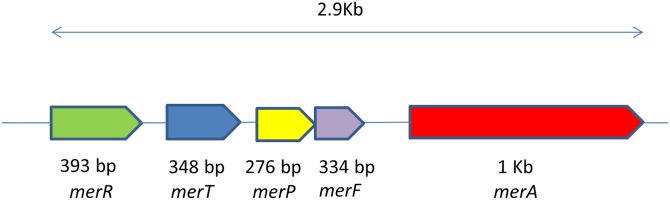
A schematic representation of the *mer* operon of MELD1 consisting of the genes *merR*, *merT*, *merP*, *merF* and *merA*.

Since MELDI showed higher mercury reductase activity as compared to MELD2 and MELD3 the whole genome was sequenced. To identify the genes involved in endophytic interaction, the *mer* operon and for dioxin degradation were also the main focus. The De-novo genome sequencing was done using Illumina Solexa technology and the *mer* operon sequence consisting of five genes, *mer*R (KJ680153), *mer*T (KJ680154), *mer*P (KJ680155), *mer*F (KJ938417) and *mer*A (KJ680156) were blasted against the NCBI database (Fig. 4). A phylogenetic tree was constructed using the *mer* operon sequence with sequence other *mer* operon, it showed a 98% sequence similarity to the *Vibrio shilonii* (WP 006069177.1) and 77% similarity to *Cycloclasticus zancles* 7-ME (YP 008372910.1) with a query coverage of 52% and 50% respectively. The draft of the whole genome sequencing was done to confirm that the mercury reductase gene (*mer*A) which was found to be 99% similar to *Vibrio shilonii*. MELD1 was organized in an operon as *merR*-*merT*-*merP*-*merF-merA*, the same order as of *Tn21* [45]. The schematic representation of *mer* operon was showed (Fig. 5). Furthermore, the *merR* is oriented the same as the other genes, similar to some Gram negative bacteria like *Pseudoalteromonas haloplanktis* and *Tenacibaculum discolor* 9A5 [46– 47].

### Plant growth promoting properties of MELD1, MELD2 and MELD3

**Fig 6:**
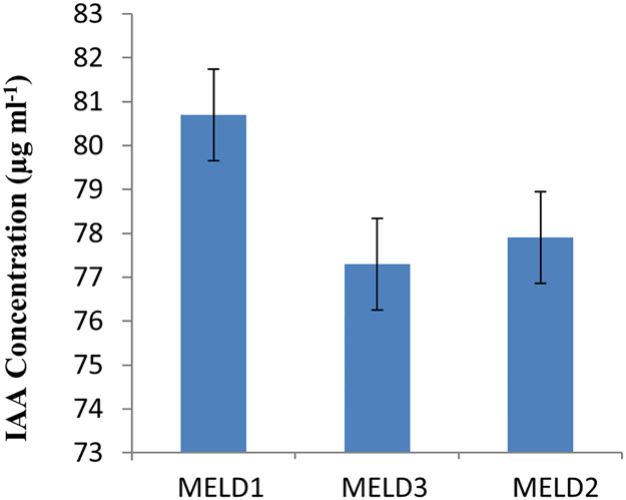
IAA production by the strains MELD1, MELD2 and MELD3. **Each value is the mean of triplicates**. Bars plot mean ± SD of three replicate experiments.

The plant growth promoting (PGP) properties like Indole 3-acetic acid were analyzed qualitatively and quantitatively (Fig. 6). The results showed that highest amount of IAA production was detected in MELD1 (81 ± 0.68 μg. ml^-1^) and then followed by MELD2 (78 ± 1.57μg. ml^-1^) and MELD3 (77 ± 1.57 μg. ml^-1^).

MELD1 being the strain with better potential as compared to MELD2 and MELD3, was chosen for phytoremediation studies. Since MELD1 showed higher mercury reductase activity and IAA production, it was chosen as a potential strain for phytoremediation studies over MELD2 and MELD3.

### MELD1, Phytoprotection and enhancing phytoremediation activity

For the phytoremediation studies, *Arabidopsis thaliana* could not be used as a model plant since they were inherently not able to survive elevated levels of mercury concentrations. This is the first research showing the application of long yard bean for phytoremediation, and was observed to grow at a mercury concentration of up to 70 mg. kg^-1^(data not shown).

**Fig 7:**
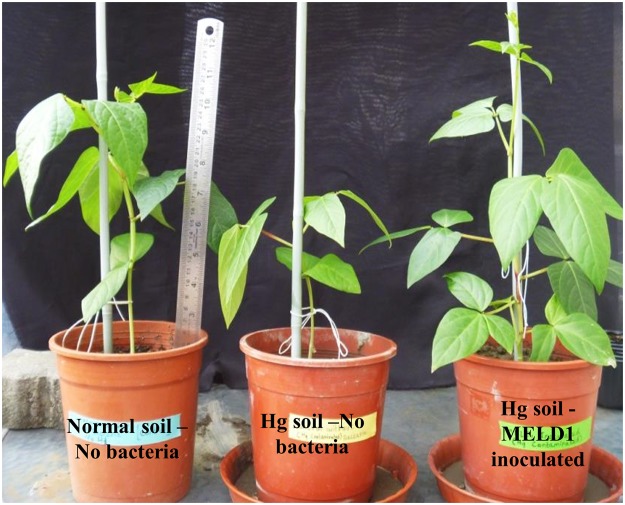
Comparison of growth between inoculated plant and control. *V*. *unguiculata* var. *sesquipedalis* grown in three different conditions; (L) grown in normal soil without bacteria, (C) grown in mercury contaminated soil without bacteria, (R) grown in mercury contaminated soil with MELD1 inoculated.

**Fig 8:**
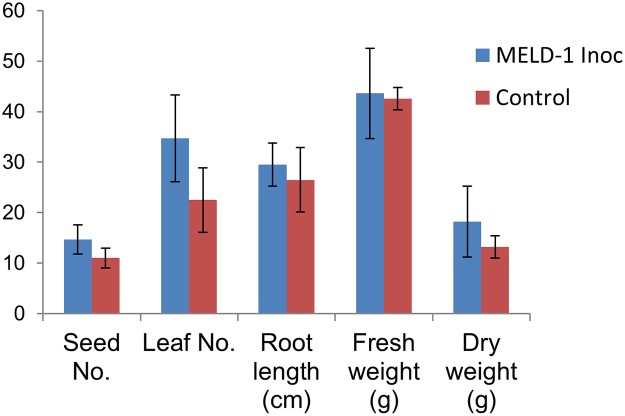
Comparing the characteristics of V. unguiculata var. sesquipedalis L. inoculated with MELD1 to the uninoculated plant. Bars plot mean ± SD of three replicate experiments.

**Fig 9:**
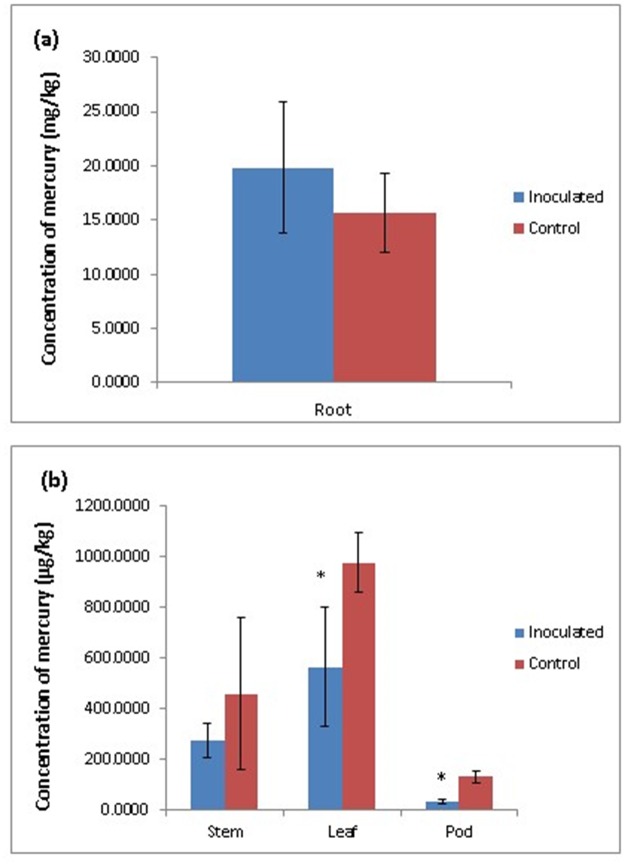
Amount of mercury accumulated in the plant parts of MELD1 inoculated bean (a) root (b) stem, leaf and pod. Bars plot mean ± SD of three replicate experiments. The * above the bar indicates significant difference among treatments at the *p<*0.05 level.

Based on the plant growth and Hg reducing property, MELD1 was selected for studying their effect on the growth of long yard bean and mercury uptake in Hg contaminated soil. It was observed that the survival and growth was greatly influenced in the plants inoculated with MELD1 rather than in uninoculated plant (Fig. 7). As seen after 3 months the MELD1 inoculated plant had a significant increase in leaf number of 54%, seed number of 33% and root length by 11%, although no significant difference was noted for the fresh weight as compared to control plants grown in contaminated soil (Fig. 8). For the uninoculated plants in the presence of mercury, the growth indices suggested that increased level of mercury resulted in phytotoxicity. Plants inoculated with MELD1, reduce mercury toxicity efficiently and showed better growth as compared to the control. After 3 months of exposure to the mercury contaminated soil, the *V*. *unguiculata* var. *sesquipedalis* was extracted and the checked for mercury concentration in various parts of plants like root, stem, leaf and pod. The concentration of mercury in *V*. *unguiculata* var. *sesquipedalis* was in the order of root>leaf>stem>pod. The concentration of mercury in the pod and leaf of the inoculated plant was significantly lower (*p<*0.05) than the uninoculated plant (Fig. 9). Highest mercury uptake was observed in the roots, but although non-significantly different, the mercury concentration in the roots of the inoculated plant was higher than that of control. It was observed that the mercury reduction in mercury contaminated soil under *in vitro* condition was comparatively lower, about 43% as compared to mercury reduction in *in vivo* conditions of about 96% (Table 3).

**Table 3:** Mercury biomineralized by MELD1 in mercury-contaminated soil.

**Trial**	**Hg Initial (mg Kg** ^-1^ **)**	**Hg Final (mg Kg** ^-1^ **)**	**Hg Biomineralization (mg Kg** ^-1^ **)**	**Hg Biomineralized (%)**
1	26.1	14.5	12.1	45.49
2	27.3	15.8	11.5	42.12
3	26.1	15.3	10.8	41.38
Mean	26.67	15.20	11.47**	43.00

$\%\text{ Hg Biomineralized}=\frac{\;\text{Initial Hg Conc }–\text{ Final Hg Conc}}{\text{Initial Hg Conc}.}\times 100$

## Discussion

Mercury is a toxic heavy metal that affects plants as well as animals. The metal is added to the soil inadvertently through fertilizer, sewage sludge and fungicides containing mercury [48]. Low cost of phytoremediation methods compared to other methods to remediate mercury, can provide a green and environment friendly method in the cleaning up of polluted soil and water. A clever solution is to combine the advantages of microbe-plant symbiosis within the plant rhizosphere into an effective cleanup technology. The effective legume-rhizosphere bacteria symbiosis in the removal of mercury has received less attention, and to our knowledge, this is the first study in mercury contaminated soil.

*V*. *unguiculata* var. *sesquipedalis* or yard long bean is an important vegetable of Kerala, India next to bitter gourd in coverage and popular preference [49]. It is a fast growing climber that grows up to a height of three to four meters and produces very long, slender and succulent pods which may be white, light green, dark green or brownish red in color. It is known that leguminous plants help to fix N_2_ into the soil. The yard long bean was thought to be evolved from cowpea (*Vigna unguiculata* L. Walp), which is native to subtropical regions. In a previous study yard long bean was seen to be demonstrating a phytostabilization potential in removing cadmium from soil [50].

In this paper, we investigated the symbiotic interaction of yard long bean and a bacterium that reduce mercury. The distribution of Hg in long yard bean inoculated with *Photobacterium* MELD1and grown in heavy metal contaminated soil was also investigated. Plant-associated bacteria have been shown to increase heavy metal extractability by plants [51–53]. Indeed, in the present study, plants inoculated with MELD1 had higher concentrations of Hg in their roots and smaller amounts in their shoots followed by leaf and a significantly lesser amount in the pods as compared to the un-inoculated plant (Fig. 9). This result is in direct correlation with result suggested by Khanna and Rai [54] and Velasco-Alinsug et. al [52]. The mercury accumulation in the leaves were a slightly higher than in the shoots; this observation is consistent with the finding of Pant et al [55] in *Impatiens walleriana*.

Previous studies using tomato, rice and oats have shown the decrease of yield when grown in mercury contaminated soil [56–58]. The work of Deivanai and Thulasyammal [50] showed that long yard bean grown in the presence of cadmium could tolerate the toxicity as well as help in the phytostabilization of cadmium and the less root to shoot translocation of cadmium make it an ideal candidate for phytostabilization. In green house study, the long yard bean inoculated with MELD1 had slightly higher seed number and had longer roots as compared to the control plant. The variations in root and shoot length could be attributed to the reduction in cell division and elongation in meristematic tissue which eventually inhibited the plant growth as well as the crop yield, due to the stress from mercury [59]

*Photobacterium* species are primarily marine organisms [60–62] but the three identified strains were isolated from the roots of a plant *Phragmites australis*, which is one of the dominant plant species in the An-shun site which is known to accumulate heavy metals [63]. *Photobacterium* is a genus of Gram-negative bacteria in the family *Vibrionaceae*. The genus *Photobacterium*, currently contains sixteen species, most of them isolated from seawater, and others from marine animals. Defined as facultative anaerobic and weakly halophilic, *Photobacterium* sp. were originally thought to be mostly luminescent, but more than a half of the recognized species do not display this ecologically important character.

Since the strains were isolated from an environment contaminated with heavy metals, they were assayed for metal tolerance. The strains MELD1, MELD2 and MELD3 were able to resist varying concentrations of Hg, Pb, Cd, PCP, PCE, CBA, TCDD and HXCDD. The annotation confirmed that MELD1 has a mercury reductase gene (*mer*A) which was found to be 99% similar to *Vibrio shilonii*. Since due to the high degree of similarity to *Vibrio shiloni*, it could be due to the horizontal gene transfer of *mer* operon between different bacterial genera [64] and that could suggest the close resemblance of *Photobacterium* to *Vibrio* species [65]. Since mercury resistance determinants are often located on mobile genetic elements such as plasmids and transposons, the mer operon can be transferred between species [66– 67]. In the present study, mercury resistance of the strain MELD1 was observed. It was detected that the *mer* operon is located on the chromosome and not on the plasmid. To our knowledge this is the first *Photobacterium* strain isolated from the terrestrial environment with high mercury resistance. MELD1 was organized in an operon as *merR*-*merT*-*merP*-*merA*, the same order (Fig. 5) as of *Tn21* [45]. Furthermore, the *merR* is oriented the same as the other genes, similar to some Gram negative bacteria like *Pseudoalteromonas haloplanktis* and *Tenacibaculum discolor* 9A5 [46– 47]. The *mer* operon had a length of 2.9 Kb and a GC content of 46 mol%. The novel resistance to mercuric chloride is conferred by the mercury reductase gene product *merA*. This enzyme *merA* reduces Hg^2+^ to less toxic elemental mercury. The *mer* operon of MELD1 was found to be 98% similar to *V*.*shilonii* AK1 with query coverage of 60%. Based on the 16S rDNA sequence homology (Fig. 1), *V*.*shilonii* strain AK1 is evolutionary related to MELD1, hence they do not share conserved inheritance of the *mer* gene. Since they belong to the same family a natural horizontal gene transfer by transposons between and among gram positive bacteria and gram negative bacteria might explain this paralogous inheritance [68–69]. Growth curve measurements showed that MELD1 attains the mid-log phase of growth at 3.5–4 hours and the stationary phase after 10 hours (S1 Fig.) with a maximal biomass accumulation of 1830 g/kg (S1 Table). The *Photobacterium* strain MELD1 showed a distinct growth pattern in the presence and absence of 25 mg kg^-1^ Hg^2+^ (S2 Fig.). The presence of Hg^2+^ decreased the initial growth rate of MELD1 extending the lag phase for one more hour. Furthermore, the strain grown in medium containing mercury reached stationary phase 3 hours earlier as compared to the strain grown in the absence of mercury. In presence of inducing concentrations of Hg^2+^, MerR activates transcription of the functional genes in *mer* operon, thereby activating *merA* converting Hg^2+^ to Hg^0^, which in turn helps the bacteria to grow efficiently in high concentrations of mercury [16]. However the growth of MELD1 in mercury was comparable to that without mercury during the exponential phase. The bioremediation potential of strain MELD1 with mercury containing LB medium was evaluated and it was seen that MELD1 had an efficiency removal rate of 96% for 25 mg kg^-1^ mercury. The mercury biomineralization assay by MELD1 in the An-shun mercury contaminated soil (Table 3) showed a lower efficiency as compared to *in-vitro* studies (Fig. 3).

The growth of *Photobacterium* as a rhizosphere-associated symbiont is an interesting study since it is not usual for a marine bacterium to be associated with terrestrial plants. The specific role of *Photobacterium* in the roots of the reed and the interaction of the two organisms in bioremediation is another area of study to be explored.

In summary, inoculation of *Photobacterium* MELD1 enhanced the growth of *V*. *unguiculata* var. *sesquipedalis* in Hg contaminated soil, increased the total Hg uptake in roots but significantly decreased Hg concentration in pods. Furthermore, inoculation of plants with PGP strains was seen to contribute in reducing the phytotoxic effects of the metals by sharing the metal load due to their ability of bioaccumulation and biosorption. Our result provides a new insight into the roles of plant-microbe symbiosis in mediating the impact of heavy metals on plants. This isolate promises a use for mercury bioremediation of contaminated soil or industrial water, and as a symbiont for plants for an increased mercury biomineralization.

## Supporting Information

S1 Fig Graph representing the growth curve of MELD1 at temperature 28^°^C in terms of optical density 600nm. (TIF)

S2 Fig Growth characteristics of MELD1 in mercury free and mercury containing medium (25 mg. kg-1) at temperature 28^°^C in terms of optical density 600nm. (TIF)

S1 Table Total biomass of MELD1. (DOCX)
