# Supplementary figures and images for: A Rhizosphere-Associated Symbiont, Photobacterium spp. Strain MELD1, and Its Targeted Synergistic Activity for Phytoprotection against Mercury
Source: PLoS One. 2015 Mar 27;10(3):e0121178. doi: 10.1371/journal.pone.0121178 (PMC4376707; doi:10.1371/journal.pone.0121178)

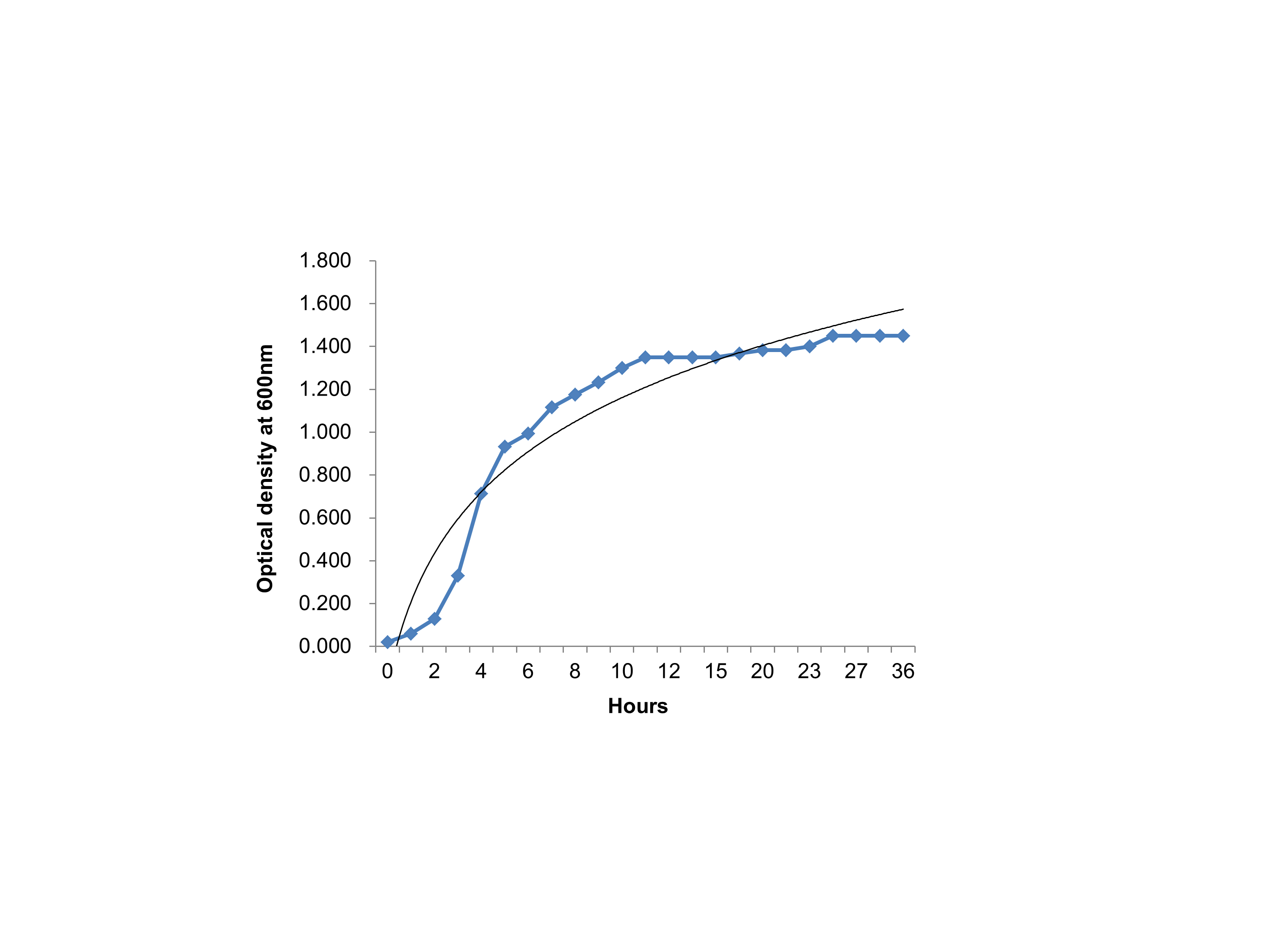

Supplement: S1 Fig — (TIF) [file pone.0121178.s001.tif]

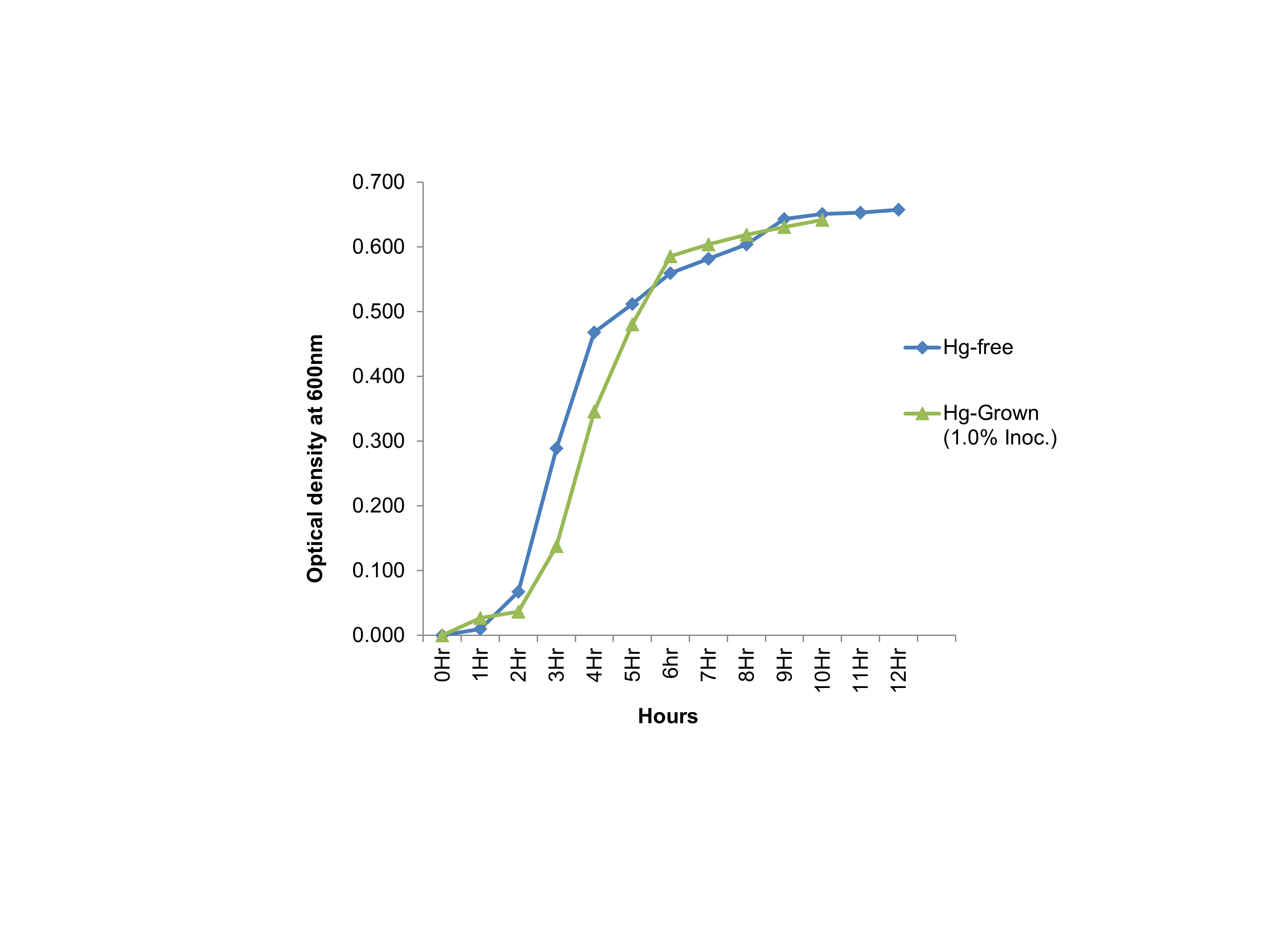

Supplement: S2 Fig — (TIF) [file pone.0121178.s002.tif]
