# Supplementary material for: A Rhizosphere-Associated Symbiont, Photobacterium spp. Strain MELD1, and Its Targeted Synergistic Activity for Phytoprotection against Mercury
Source: PLoS One. 2015 Mar 27;10(3):e0121178. doi: 10.1371/journal.pone.0121178 (PMC4376707; doi:10.1371/journal.pone.0121178)
Supplement: S1 Table — (DOCX) [file pone.0121178.s003.docx]

|  |  |  |  | **Constant Dry weight** | |  |
| --- | --- | --- | --- | --- | --- | --- |
| **Rep** | **Weight of tube (mg)** | **Weight of tube with cells (mg)** | **Wet Weight (mg)** | **Dry weight 1 (mg)** | **Dry weight 2 (mg)** | **Dry Weight (mg)** |
| 1 | 9936 | 9983 | 47 | 9953 | 9953 | 17.00 |
| 2 | 9922 | 9972 | 50 | 9940 | 9940 | 18.00 |
| 3 | 9916 | 9964 | 48 | 9933 | 9934 | 18.00 |
| 4 | 9853 | 9896 | 43 | 9871 | 9873 | 20.00 |
| 5 | 9888 | 9934 | 46 | 9903 | 9906 | 18.00 |
| 6 | 9950 | 9995 | 45 | 9966 | 9969 | 19.00 |

**Biomass = Constant dry weight – weight of the tube**

**Volume of the sample**
